# Subgroup analysis reveals higher reliability of the new comprehensive evaluation of Global Initiative for Chronic Obstructive Lung Disease 2019

**DOI:** 10.1038/s41598-021-04756-w

**Published:** 2022-01-14

**Authors:** Zhongshang Dai, Huihui Zeng, Yanan Cui, Ping Chen, Yan Chen

**Affiliations:** Second Xiangya Hospital of Central South University, Changsha, China

**Keywords:** Chronic obstructive pulmonary disease, Preventive medicine

## Abstract

To estimate the severity of the disease in outpatients with chronic obstructive pulmonary disease (COPD) in Hunan Province, China and use the subgroup analysis to evaluate the reliability of the new comprehensive evaluation of Global Initiative for Chronic Obstructive Lung Disease (GOLD). COPD outpatients from 12 medical centers in Hunan Province, China were stratified into groups A–D, and group D patients were further stratified into subgroups D_1_–D_3_ according to the GOLD 2016 and 2019 comprehensive assessment. Demography, clinical characteristics and medications were compared among groups. In 1017 COPD outpatients, the distribution from group A to D and subgroup D_1_ to D_3_ was 41 (4.0%), 249 (24.5%), 17 (1.7%), 710 (69.8%) and 214 (30.2%), 204 (28.7%), 292 (41.1%), according to GOLD 2016. In terms of demographic and clinical characteristics related to A–D groups, there was a significant difference in COPD assessment test (CAT), modified Medical British Research Council (mMRC), the clinical COPD questionnaire(CCQ), age, BMI, education level, smoking history, comorbidities, the course of chronic bronchitis/emphysema, number of exacerbations/hospitalisations in the previous year, treatment protocols, forced expiratory volume in one second (FEV1) % predicted, and FEV1/forced vital capacity (FVC) (*p* < 0.01). Furthermore, some patients in groups C–D regrouped to groups A–B were all C_1_ and D_1_ subgroups according to GOLD 2019. Comparing subgroup D_1_ with group B, subgroup D_2_ and subgroup D_3_, it was found that the demography, clinical characteristics and medications of subgroup D_1_ were the closest to group B, according to GOLD 2016 (*p* < 0.01). The disease severity of outpatients with COPD in Hunan Province was more pronounced in group B and D and patients in groups A–D had different demography, clinical characteristics and medications. Subgroup analysis can explain to a certain extent that GOLD2019’s new comprehensive assessment is more reliable than GOLD 2016.

## Introduction

Chronic obstructive pulmonary disease (COPD) is a global public health challenge due to its high prevalence and related mortality^1^. The most recent China Pulmonary Health (CPH) study reported the overall prevalence of spirometry-defined COPD was 8.6% among the general Chinese population aged 20 years or older and the estimated total number of individuals was 99.9 million^2^. However, no Hunan Province data are available for severity of COPD outpatients.

The clinical management of COPD was mostly guided by the Global Initiative for Chronic Obstructive Lung Disease (GOLD) document. From GOLD 2011 to GOLD 2016, patients were stratified by ABCD assessment tool, which incorporated symptoms, spirometry measure, and frequency of exacerbations. Lange et al. further performed analyses of the subgroups of the C and D categories, as patients can be stratified into these categories through different scenarios^3^. Thus, categories C and D were subdivided into subgroups C_1_, C_2_, C_3_, D_1_, D_2_, and D_3_. They found groups C and D are heterogeneous, being composed of phenotypes with variable risk. In the 2019 update of GOLD document, a refinement of the ABCD assessment tool was proposed that separated spirometry measure from the ABCD group^4^. Because of the brand new assessment tool, some patients in groups C–D were regrouped to groups A–B. However, we are not sure whether the refined ABCD assessment scheme is more suitable for the COPD population grouping than the previous creterion.

In this study, we aimed to estimate the severity of the disease in outpatients with COPD in Hunan Province, China. Furthermore, we use the subgroup analysis to evaluate the reliability of GOLD 2019.

## Methods

### Study participants

The study was a cross-sectional observational survey in Hunan Province, China. From January 2017 to January 2018, patients were recruited from COPD outpatient clinics from 12 tertiary hospitals in Hunan Province, China. The study was approved by the ethics review committee of the Second Xiangya Hospital, Central South University and the ethics registration number was: No. ChiCTR-POC-17010431. We got informed consent from all study participants. All methods including the diagnosis of COPD and spirometry test were performed in accordance with COPD guidelines and regulations^4^.

Patients who were recruited had a clear diagnosis of COPD according to the GOLD 2019, based on the persistent airflow limitation defined as post-bronchodilator forced expiratory volume in one second (FEV1)/forced vital capacity (FVC) < 70%. Furthermore, the patients were in a stable stage, that was, there was no acute exacerbation within one month and they were proficient in Chinese with no communication barriers. Patients suffering from other diseases that cause airflow limitation or decreased lung capacity were excluded.

### Study procedure

We performed the study in the form of questionnaires, including a self-made questionnaire, COPD Assessment Test (CAT) questionnaire, modified Medical British Research Council (mMRC) dyspnoea scale, and the clinical COPD questionnaire (CCQ). Face-to-face interviews were conducted among the included patients by specially-assigned persons. The self-made questionnaire included gender, age, BMI, COPD family hiotory, education level, occupational exposure history, biofuel exposure history, smoking history, smoking pack-years, comorbidities, the course of chronic bronchitis/emphysema, number of exacerbations in the previous year, number of hospitalisations in the previous year, treatment protocols, FEV1% predicted, and FEV1/FVC.

The severity of airflow limitation categories was defined according to GOLD 2007: I (mild): FEV1 ≥ 80% predicted; II (moderate): 50% ≤ FEV1 < 80% predicted; III (severe): 30% ≤ FEV1 < 50% predicted; IV (very severe): FEV1 < 30% predicted. An exacerbation was defined as 1.worsening of symptoms beyond normal day-to-day variations that required additional treatment with oral or intravenous corticosteroids, antibiotics, or both for an intended duration of ≥ 3 days; 2.attendance at an emergency center for worsening of symptoms; or 3.a hospital admission with a primary diagnosis of COPD. Evaluation of symptoms was based on mMRC scale or CAT scores to indicate whether the patients has fewer symptoms (mMRC grade 0–1 or CAT score < 10) or more symptoms (mMRC grade ≥ 2 or CAT score ≥ 10). The worse of the two evaluations was considered in the classification. Exacerbation risk was assessed with airflow limitation measured by postbronchodilator FEV1% predicted (< 50% or ≥ 50%) or the number of COPD exacerbations in the previous year (≤ 1 or ≥ 2). Of note, at least one hospitalization for a COPD exacerbation during the past year was considered as high risk. When the two evaluations were inconsistent, the assessment indicating higher risk was used. The GOLD 2016 comprehensive assessment classified patients in categories of A (low risk, fewer symptoms), B (low risk, more symptoms), C (high risk, fewer symptoms), and D (high risk, more symptoms). However, the GOLD 2019 assessment abolished the degree of airflow limitation from the grading system and exacerbation risk was assessed only by exacerbation history in the previous year, which stratified patients into low-risk categories (A and B) and high-risk categories (C and D). The methods of assessing symptoms remained unchanged^1,3,4^.

### Statistical analysis

The data were analysed using Statistical Package for Social Sciences (SPSS) version 21.0 and R software version 3.6.2 (R Foundation for Statistical Computing). The data in this study were non-normally distributed after the normality test. Descriptive data without normal distribution were expressed as medians (interquartile range [IQR]), and frequencies were expressed as numbers (percentage). The Wilcoxon and Kruskal–Wallis H tests were used to compare the A, B, C, D groups and D subgroups. Forest plot using standardized mean difference of measurement data and odds ratio of counting data was used to compare subgroup D_1_ with group B, subgroup D_2_ and subgroup D_3_. Statistical significance was set at *p* < 0.05.

## Results

### Demographic characteristics

**Figure 1:**
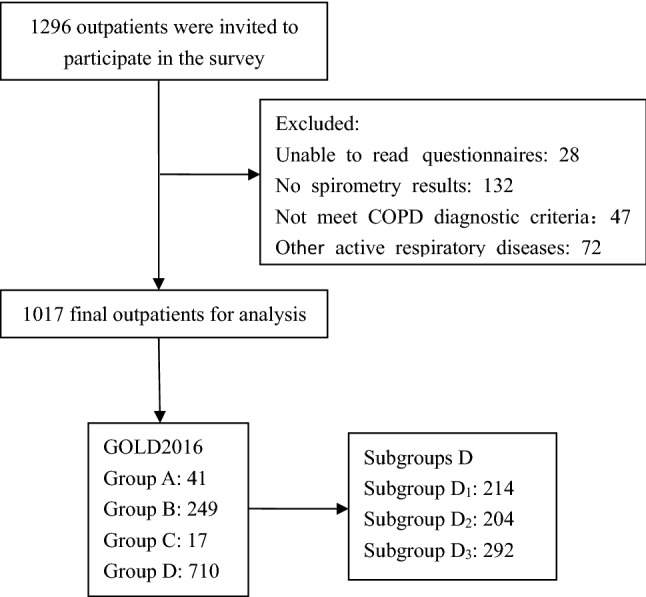
Study flow chart. *COPD* Chronic obstructive pulmonary disease, *GOLD* Global Initiative for Chronic Obstructive Lung Disease. D_1_: Forced expiratory volume in one second (FEV1) < 50% predicted and fewer than two exacerbations (and < 1 hospitalized exacerbation) in the previous year. D_2_: FEV1 ≥ 50% predicted and two or more exacerbations (or ≥ 1 hospitalized exacerbation ) in the previous year. D_3_: FEV1 < 50% predicted and two or more exacerbations (or ≥ 1 hospitalized exacerbation) in the previous year.

From the original cohort including 1296 outpatients, 279 patients were excluded. Of these, 28 patients were unable to read the questionnaire, 132 patients had no spirometry results, 47 patients did not meet the COPD diagnostic criteria, and 72 patients had other active respiratory diseases. Finally, a total of 1017 outpatients meeting the study criteria were included (Fig. 1).

**Figure 2:**
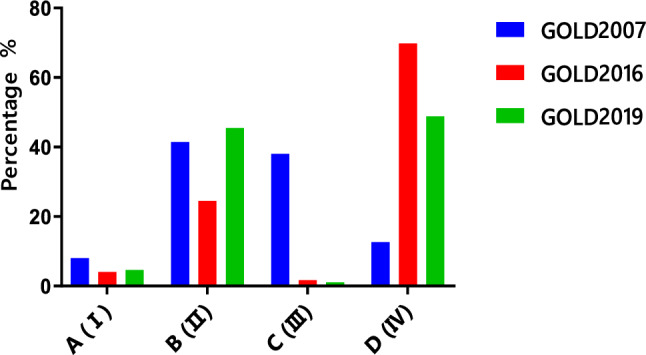
Comparison of the distribution of COPD patients using the GOLD groups A–D/I–IV 2007, 2016 and 2019 classifications. *GOLD* Global Initiative for Chronic Obstructive Lung Disease.

Of the 1017 outpatients recruited to this study, the distribution of comprehensive assessment groups according to GOLD 2007: Grade I was 8.0% (81/1017), Grade II was 41.4% (421/1017), Grade III was 38.0% (387/1017), and Grade IV was 12.6% (128/1017). Using the GOLD 2016 comprehensive assessment, group A was 4.0% (41/1017), B was 24.5% (249/1017), C was 1.7% (17/1017), and D was 69.8% (710/1017). Using the GOLD 2019 comprehensive assessment, group A was 4.6% (47/1017), B was 45.5% (463/1017), C was 1.1% (11/1017), and D was 48.8% (496/1017) (Fig. 2). Therefore, the disease severity of outpatients with COPD in Hunan Province was more common in group B and D. Group D patients were further stratified into subgroups D_1_–D_3_ according to the GOLD 2016: D_1_ was 30.2% (214/710), D_2_ was 28.7% (204/710), D_3_ was 41.1% (292/710). Group C patients were unable to perform subgroup analysis and statistical analysis because of small sample sizes.

**Figure 3:**
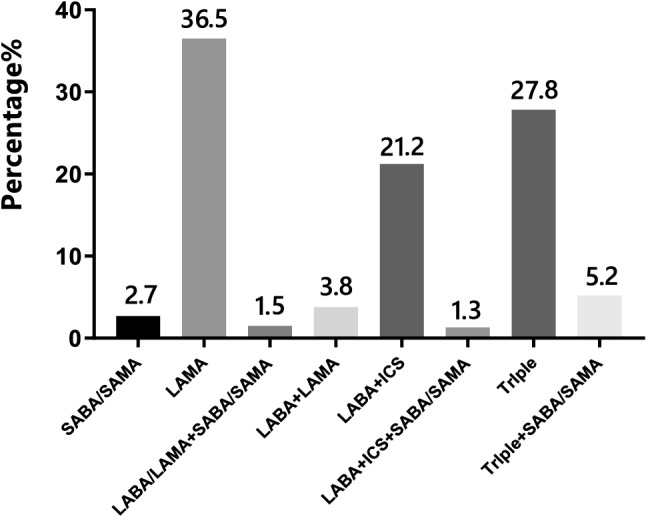
Comparison of the distribution of prescriptions in 1017 patients with COPD. *SABA* Short-acting beta-agonist, *SAMA* Short-acting muscarinic receptor agonist, *LAMA* Long-acting muscarinic receptor agonist, *LABA* Long-acting beta-agonist, *ICS* Inhaled corticosteroids, Triple, LAMA + LABA + ICS.

**Figure 4:**
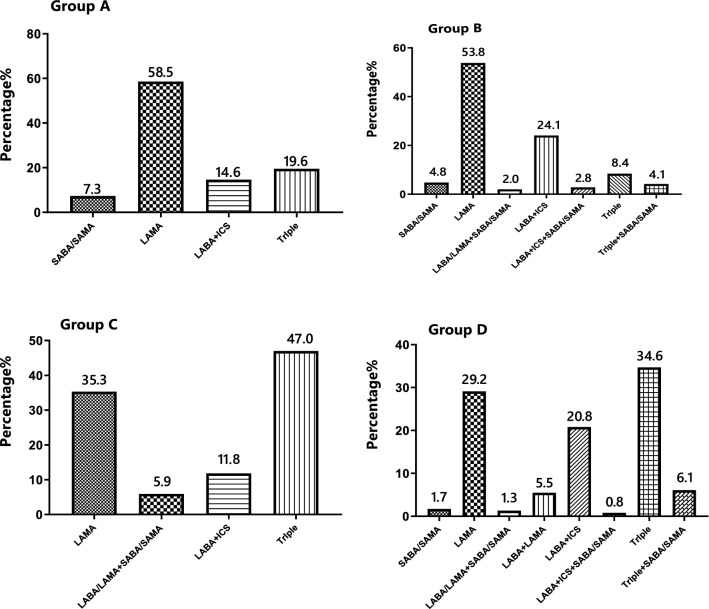
Comparison of the distribution of prescriptions in1017 patients with COPD in groups A–D.

In the groups A, B, C, and D, there was no significant difference in gender, family history of COPD, history of exposure to biofuel, and smoking index. The proportion of high-education (12.2%), FEV1/FVC (65.0%), and FEV1% predicted (82.0%) in group A was significantly higher than that in group B, C, and D, while the occupational exposure history (19.5%), the course of disease (1 years), and questionnaire (CAT, mMRC and CCQ) scores were significantly lower than those in groups B, C, and D. The proportion of current smoking patients (81.1%), BMI index (25.2 kg/m^2^), and the proportion of coronary artery disease (19.3%) in group B were the highest, and the proportion of former-smokers (4.4%) was the lowest among the groups A, B, C, and D. Patients in group C had no specific clinical features and the lowest proportion. On the contrary, the course of disease (13 years), exacerbations in the previous year (2 times), and questionnaire scores were significantly higher in the D group than in the A, B, and C groups, and FEV1/FVC (42.0%), FEV1% predicted (43.7%) were significantly lower than those in the A, B, and C groups (*p* < 0.01) (Table 1). The most frequently prescribed drugs were single-inhaler long-acting muscarinic antagonist (LAMA)-only treatment (36.5%), followed by triple inhaled treatment (27.8%) with an inhaled corticosteroids (ICS) plus long-acting beta-agonist (LABA) and LAMA. A total of 564 patients (55.5%) were treated with ICS (Fig. 3). Furthermore, the most commonly treatment protocol was single-inhaler LAMA in group A (58.5%) and group B (53.8%), while the most frequently prescribed drug was triple inhaled treatment in group C (47.0%)and group D (34.6%) (Fig. 4).

**Table 1:** The distribution by GOLD A-D comprehensive assessment in demographic and clinical characteristics.

Item	Group A	Group B	Group C	Group D	Statistics	*p* value
Total number, n (%)	41 (4.0%)	249 (24.5%)	17 (1.7%)	710 (69.8%)		
Male patients, n (%)	33 (80.5%)	206 (82.7%)	14 (82.4%)	619 (87.2%)	4.13	0.247
Age (years)*	62 (14.5)	64 (12.5)	60 (13.5)	65 (11.0)	11.89	0.008
BMI (kg/m^2^)*	21.5 (4.3)	25.2 (4.7)	19.5 (3.9)	21.5 (4.6)	161.98	0.000
COPD family history, n (%)	5(12.2%)	33(13.3%)	2(11.8%)	139(19.6%)	6.37	0.095
Education level, n (%)					16.28	0.001
Primary school and below	13 (31.7%)	134 (53.8%)	8 (47.1%)	389 (54.8%)		
Junior high school	11 (26.8%)	75 (30.1%)	4 (23.5%)	226 (31.8%)		
High school	12 (29.3%)	35 (14.1%)	5 (29.4%)	71 (10.0%)		
University and above	5 (12.2%)	5 (2.0%)	0 (0.0%)	24 (3.4%)		
Occupational exposure history, n (%)	8 (19.5%)	71 (28.5%)	6 (35.3%)	255 (35.9%)	8.26	0.041
Biofuel exposure history, n (%)	10 (24.4%)	63 (25.3%)	3 (17.6%)	192 (27.0%)	1.06	0.787
Smoking history, n (%)					25.73	0.000
Never-smokers	5 (12.1%)	36 (14.5%)	0 (0.0%)	76 (10.7%)		
Former-smokers	9 (22.0%)	11 (4.4%)	9 (52.9%)	212 (29.9%)		
Current smokers	27 (65.9%)	202 (81.1%)	8 (47.1%)	422 (59.4%)		
Smoking pack-years*	30.0 (40.0)	30.0 (50.0)	30.0 (42.5)	30.0 (40.0)	2.66	0.447
Comorbidities, n (%)						
Coronary artery disease	4 (10.2%)	48 (19.3%)	2 (11.8%)	94 (13.2%)	15.13	0.001
Diabetes mellitus	4 (10.2%)	29 (11.6%)	2 (11.8%)	69 (9.7%)	1.20	0.721
The course of chronic bronchitis/emphysema (years)*	1.0 (0.6)	6.0 (0.0)	4.0 (0.0)	13.0 (8.0)	619.23	0.000
Hospitalizations in the previous year*	0.0 (0.0)	0.0 (0.0)	0.0 (1.0)	0.0 (1.0)	209.28	0.000
Exacerbations in the previous year*	0.0 (0.0)	0.0 (1.0)	1.0 (2.0)	2.0 (2.0)	113.57	0.000
FEV1% predicted*	82.0 (19.8)	64.8 (16.0)	45.5 (42.3)	42.0 (19.5)	357.49	0.000
FEV1/FVC*	65.0 (7.8)	58.0 (13.2)	53.0 (28.9)	43.7 (19.0)	236.15	0.000
mMRC*	0.0 (1.0)	1.0 (1.0)	1.0 (1.0)	2.0 (2.0)	157.15	0.000
CAT*	7.0 (3.5)	16.0 (5.0)	8.0 (2.0)	18.0 (6.0)	197.29	0.000
CCQ*	9.0 (6.0)	24.0 (7.0)	15.0 (5.5)	27.0 (7.0)	56.91	0.000

*BMI* Body mass index, *FEV1* Forced expiratory volume in 1 s, *FVC* Forced vital capacity, *CAT* COPD assessment test, *mMRC* Modified Medical British Research Council, *CCQ* Clinical COPD questionnaire, *IQR* Interquartile range.

*Non-normally distributed data and data are shown as median [interquartile range (IQR)].

**Figure 5:**
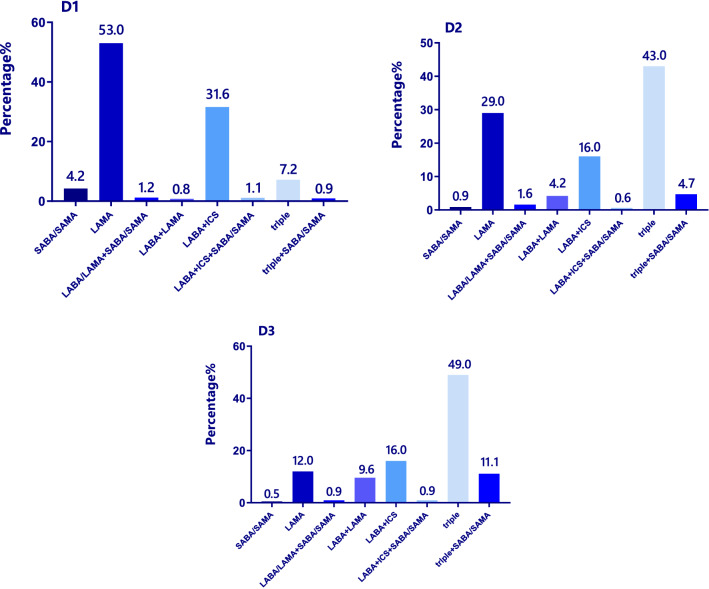
Comparison of the distribution of prescriptions in 1017 patients with COPD in Subgroups D1, D2, D3.

In the subgroups D_1,_ D_2,_ and D_3_, there was no significant difference in age, family history of COPD, history of exposure to biofuel, education level, CCQ score and occupational exposure history. The proportion of current smoking patients (67.3%), BMI index (21.8 kg/m^2^), and the proportion of coronary artery disease (17.3%) in subgroup D_1_ were the highest, whereas the proportion of former-smokers (23.2%) was the lowest among the subgroups D_1,_ D_2,_ and D_3_. Besides, the exacerbations in the previous year (0 time) was significantly lower in subgroup D_1_ than in subgroups D_2_, D_3_ (Table 2). There is a large difference in the proportion of drug prescriptions among patients in the D subgroups. the most commonly treatment protocol was single-inhaler LAMA (53.0%) in subgroup D_1_, while the most frequently prescribed drugs were triple inhaled treatment in subgroup D_2_ (43.0%) and subgroup D_3_ (49.0%) (Fig. 5).

**Table 2:** The distribution by GOLD subgroups D comprehensive assessment in demographic and clinical characteristics.

Item	Subgroup D1	Subgroup D2	Subgroup D3	Statistics	*p* value
Total number, n (%)	214 (30.2%)	204 (28.7%)	292 (41.1%)		
Male patients, n (%)	196 (91.6%)	159 (77.9%)	264 (90.4%)	22.00	0.000
Age (years)*	64.0 (11.0)	65.0 (11.0)	65.0 (8.0)	1.89	0.388
BMI (kg/m^2^)*	21.8 (4.4)	21.2 (4.8)	21.3 (4.7)	6.03	0.049
COPD family hiotory, n (%)	41 (19.2%)	43 (21.1%)	55 (18.8%)	0.41	0.812
Education level, n (%)				1.43	0.489
Primary school and below	119 (55.6%)	118 (57.8%)	152 (52.1%)		
Junior high school	57 (26.6%)	63 (30.9%)	106 (36.6%)		
High school	29 (13.6%)	18 (8.8%)	24 (8.2%)		
University and above	9 (4.2%)	5 (2.5%)	10 (3.4%)		
Occupational exposure history, n (%)	71 (33.2%)	84 (41.2%)	100 (34.2%)	3.49	0.174
Biofuel exposure history, n (%)	47 (22.0%)	56 (27.5%)	89 (30.5%)	4.55	0.102
Smoking history, n (%)				9.19	0.010
Never-smokers	14 (6.5%)	33 (16.2%)	29 (9.9%)		
Former-smokers	56 (26.2%)	55 (27.0%)	101 (34.6%)		
Current smokers	144 (67.3%)	116 (56.9%)	162 (55.5%)		
Smoking pack-years*	30.0 (33.5)	30.0 (40.0)	30.0 (36.9)	6.63	0.036
Comorbidities, n (%)					
Coronary artery disease	37 (17.3%)	21 (10.1%)	33 (11.2%)	15.23	0.001
Diabetes mellitus	24 (11.1%)	17 (8.3%)	28 (9.5%)	6.20	0.047
The course of chronic bronchitis/emphysema (years)*	10.0 (0.0)	15.0 (3.0)	18.0 (5.0)	271.58	0.000
Hospitalizations in the previous year*	0.0 (0.0)	1.0 (1.0)	1.0 (2.0)	272.31	0.000
Exacerbations in the previous year*	0.0 (1.0)	1.0 (2.0)	2.0 (2.8)	128.82	0.000
FEV1% pred*	39.2(12.6)	62.9(17.6)	34.1(13.9)	444.03	0.000
FEV1/FVC*	39.8(12.5)	56.0(15.0)	38.0 (15.2)	246.04	0.000
mMRC*	2.0 (2.0)	2.0 (2.0)	2.0 (1.0)	33.84	0.000
CAT*	17.0 (4.0)	18.0 (6.0)	19.0 (4.8)	30.38	0.000
CCQ*	27.0 (6.3)	27.0 (8.0)	28.0 (7.0)	2.31	0.314

*BMI* Body mass index, *FEV1* Forced expiratory volume in 1 s, *FVC* Forced vital capacity, *CAT* COPD assessment test, *mMRC* Modified Medical British Research Council, *CCQ* Clinical COPD questionnaire, *IQR* Interquartile range.

*Non-normally distributed data and data are shown as median [interquartile range (IQR)].

### Comparison between ABCD group and D subgroup

**Figure 6:**
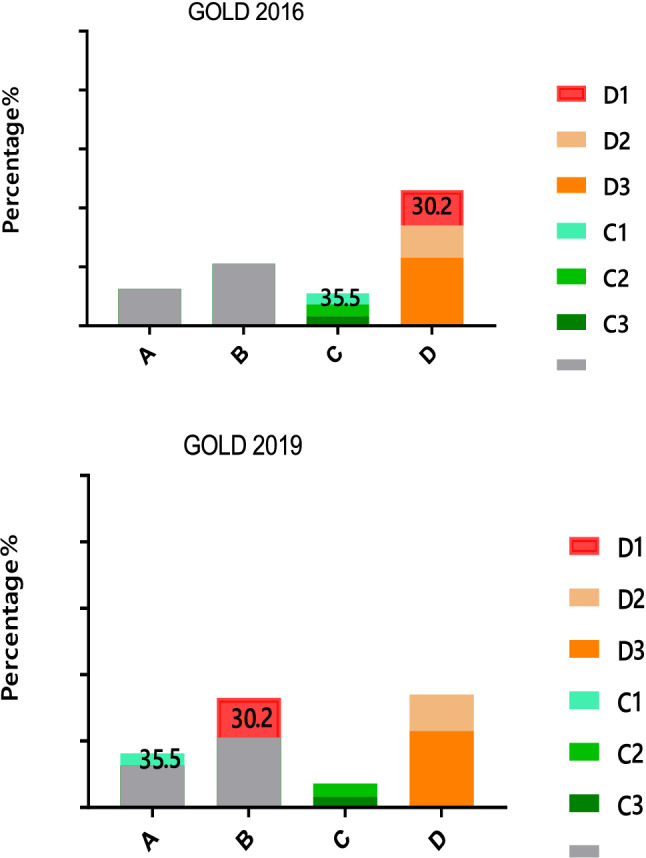
Comparison of the distribution and transferring of COPD patients in the A-D group and the C/D subgroups in GOLD 2016 and 2019 classifications. *GOLD* Global Initiative for Chronic Obstructive Lung Disease.

**Figure 7:**
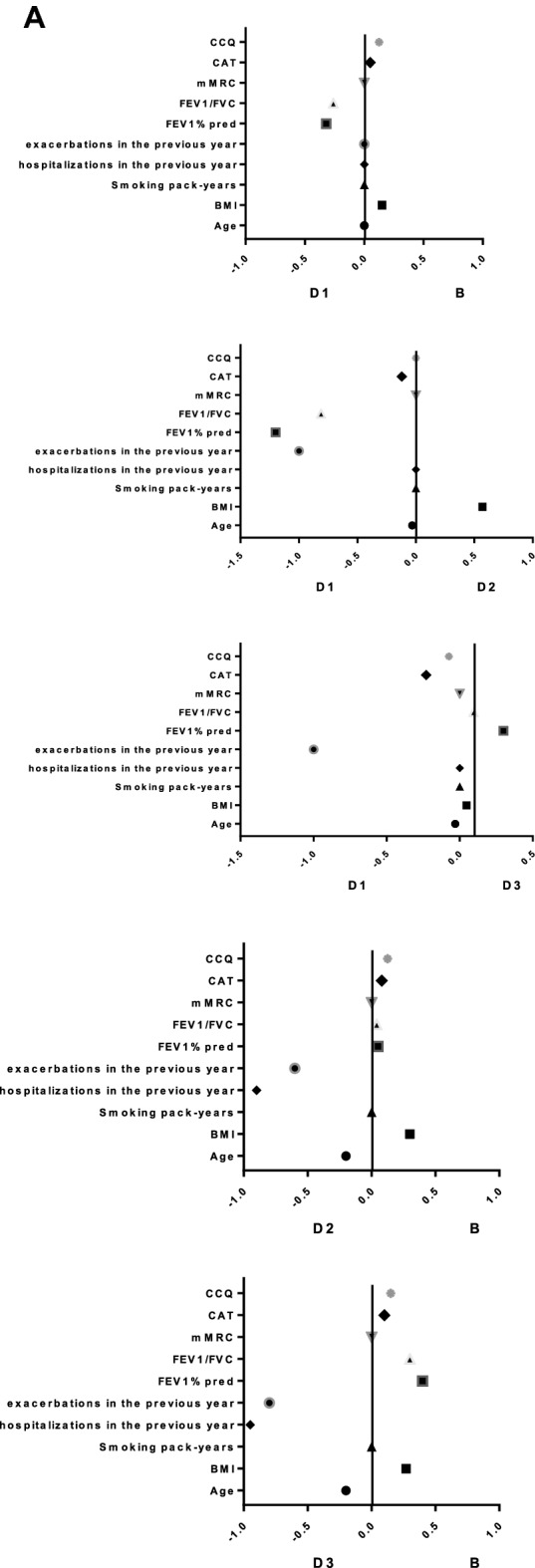

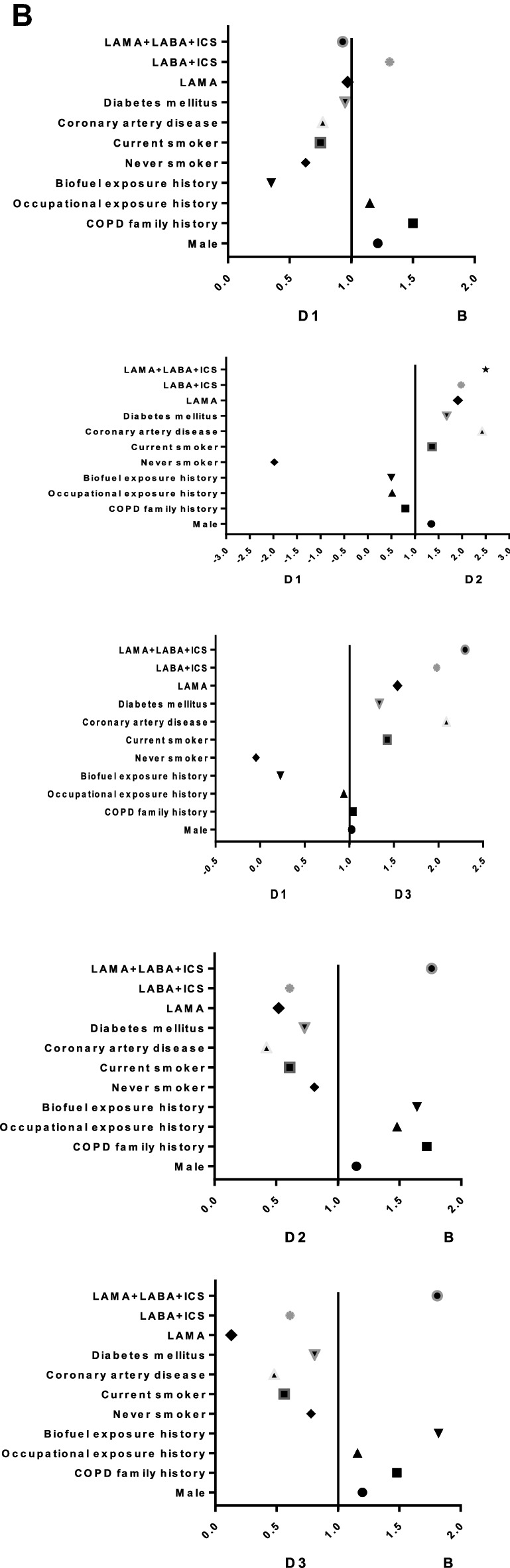
Comparison of demographic, clinical characteristics and medications between subgroup D1, group B, subgroup D2, and subgroup D3. *Notes* (**A**) Forest plot using standardized mean difference of demographic, clinical characteristics and medications for subgroup analysis. (**B**) Forest plot using odds ratio of demographic, clinical characteristics and medications for subgroup analysis.

According to the comprehensive assessment of the new GOLD2019 document, some patients in groups C–D were regrouped to groups A–B, especially 214 patients (30.2%) in group D were regrouped to group B. However, in our study, we found that the groups C and D regrouped to groups A and B were all subgroups C_1_ and D_1_: 6 patients (35.3%) in subgroup C_1_ were regrouped to group A, and 214 patients (30.2%) in subgroup D_1_ were regrouped to group B, which suggested that patients in the subgroups C_1_ and D_1_ may have the similar demographic and clinical characteristics as patients in groups A and B (Fig. 6). By further comparing the groups B, subgroup D_1_, subgroup D_2_ and subgroup D_3_, it was found that the demography, clinical characteristics and medications of subgroup D_1_ were the closest to group B, especially in the proportion of current smoking patients, BMI index, coronary artery disease, former-smokers, and treatment protocol (*p* < 0.01) (Fig. 7).

## Discussion

To the best of our knowledge, this study is the first survey focusing on the distribution, clinical characteristics and medications of COPD patients recruited from the outpatient clinics in Hunan, China. At the same time, we are committed to use the subgroup analysis to evaluate the changes of definition in assessment of GOLD stratification 2019. In our study we found that the disease severity of outpatients with COPD in Hunan Province was more common in group B and D, especially in group D. This consequence was similar to that reported in the previous COPD case-cohort studies recruited from hospital clinics^5–7^, but was obviously different from the situation dominated by group A in COPD patients identified in the general population^3,8,9^. This result may be considered because patients who came to the hospital for medical help often had more serious symptoms and a longer course of disease, meanwhile most patients with early COPD were unaware of their condition and few had performed a previous pulmonary function test, let alone saw a doctor. So the distribution of patient categories in an area may be substantially different based on different populations and patient selection criteria. It also suggested that epidemiological studies were of great significance for the prevention and early detection of COPD using spirometry, which should be a public health priority in Hunan Province.

As we knew, the core change of refinement of the brand new ABCD assessment tool was the shift of removing spirometry measure and leaving the symptoms and frequency of exacerbations in the categorization, that brought about some patients from high-risk groups without an exacerbation history in the previous year shifting to the low-risk groups. As a result, when classified by the GOLD 2019 assessment, we found that in Cabrera and Lina Sun’s national large-scale cross-sectional studies, they showed more than one third of high-risk groups were regrouped to low-risk groups and group A occupied the largest proportion of patients^10,11^. However, group D was still the biggest group in our study, although the proportion of patients in group B was significantly higher than before, according to the revised 2019 ABCD classification. Tudoric N also presented the similar result that group B and D were the most prevalent groups in their POPE cohort^12^. This phenomenon was considered because the group D in our province had a large base number, and group C was the smallest. Even if one third of patients were reclassified, the proportion of group composition was not changed, but the proportion of patients in group D had a significant downward trend compared with the group document of GOLD 2016.

In our analyses, we observed that outpatients in group A had a short course of disease and the highest proportion of high-education. This is similar to the results of previous literature studies. A study from Poland divided patients into low, medium, and high levels of education based on the number of years of education. The risk of COPD decreased by about 35% for each level of education increase, while group A patients were mainly in the higher level of education^13^. CPH study also found that only about 10% of interviewees knew about COPD, and less than 3% of patients knew they had it. It is pointed out that low education level is an important factor that should not be ignored among many related factors of COPD disease^2^. The proportion of current smoking patients, BMI index, and the proportion of coronary artery disease in group B were the highest. In some studies, BMI has been identified as an important risk factor for COPD, with higher BMI associated with higher airway limitation^14–16^. Agusti A also found that inflammatory indicators such as C-reactive protein in group B were significantly higher than those in other groups, and the reason for severe systemic symptoms in group B may be related to systemic inflammatory response^6^. However, inflammatory factor specimens were not collected for comparative analysis in our study. The proportion of patients in group C was the lowest, which was consistent with the results of previous clinical epidemiological studies^9,17^, suggesting that patients with poor lung function and mild respiratory symptoms were rare. On the contrary, the course of disease, age, exacerbations in the previous year and questionnaire scores in group D were significantly higher than those in groups A, B and C, and the FEV1% predicted was significantly lower than that in groups A, B and C. This suggests that the risk and severity of symptoms in group D are related to the course of chronic bronchitis/emphysema, lung function, and age. Several foreign clinical studies have also suggested that age is closely related to progressive decline in lung function and frequent acute exacerbations^18,19^. Therefore, for patients with a long course of disease, progressive decline in lung function may be difficult to avoid, and proactive prevention of acute exacerbations is a key measure. Another interesting finding was that the treatment protocols in our outpatients were not exactly consistent with the GOLD guidelines. In our study, majority of the participants were treated with ICS. GOLD 2019 suggests an escalation to triple treatment only in patients of group D who develop further exacerbations (CAT ≥ 20 or blood eosinophils ≥ 300/ul) on LAMA + LABA^4^. Similarly, overtreatment has been found in increasing number of clinical studies^12,20,21^. In addition, the latest related research found that compared with LAMA alone and ICS + LABA alone, LAMA + LABA consistently demonstrated improved lung function across age and airflow limitation severity subgroups, and was particularly safe and effective in elderly patients with COPD and patients with severe airflow restriction^22^. Another real-world COPD treatment study have made the conclusion that the triple treatment is generally as effective as LAMA + LABA in preventing COPD exacerbations. However, a LAMA + LABA combination without ICS is associated with fewer severe cases of pneumonia^23^. However, only a small percentage of group D patients received a LAMA + LABA combination in our study. One major reason was that the LAMA + LABA was rarely available in most hospitals in Hunan and many physicians were more likely to use LAMA or ICS + LABA based on their empirically clinical judgment rather than GOLD guidelines.

In our study, we also observed that the demography, clinical characteristics and medications of subgroup D_1_ were the closest to group B, especially in the proportion of current smoking patients, BMI index, coronary artery disease, former-smokers, and treatment protocol. It’s not hard to see that it was actually more appropriate to stratify subgroup D_1_ into group B and the GOLD2019’s new comprehensive assessment is more reasonable and reliable than GOLD 2016. In the study, Lange et al. (2012) found that the subgroup D_1_ had a lower risk of future acute exacerbations than the other subgroups, and the subgroup D_1_ had a lower 3-year mortality rate than the subgroups D_2_ and D_3_^3^. Similarly, Han MK found in COPD gene study that there was a significant difference in acute exacerbation frequency among subgroups D, and subgroup D_3_ was the highest (1. 86 times per year), followed by subgroup D_2_ (1. 34 times per year) and subgroup D_1_ (0. 89 times per year)^9^. Therefore, it can be seen that the clinical characteristics of each subgroup in group D are very heterogeneous, so they cannot be put together for unified treatment. Reclassification of some subgroup D_1_ patients to group B is more conducive to clinical medication and prognosis judgment.

This study has some limitations. First, since this study was restricted to outpatients from 12 outpatient clinics of tertiary hospitals, the results should be generalised with caution. However, we believe that our findings have important clinical implications and objectively evaluated the situation in Hunan, reflecting the current clinical characteristics and prescribing status of COPD patients. Secondly, there were only a few patients in group A and C, which may affect the accuracy and reliability of our statistical analysis, but we reflected the real disease severity of COPD patients in Hunan Province. Thirdly, future studies should focus on other factors that may impact the characteristics of COPD subgroups.

## Conclusion

The disease severity of outpatients with COPD in Hunan Province was more pronounced in group B and D and patients in groups A–D had different demography, clinical characteristics and medications. Subgroup analysis can explain to a certain extent that GOLD2019’s new comprehensive assessment is more reliable than GOLD 2016.
